# Urinary Kim-1 Correlates with Interstitial Nephritis Activity in Patients with Microscopic Polyangiitis

**DOI:** 10.3390/cimb47030196

**Published:** 2025-03-16

**Authors:** Chisato Ashida, Yuji Nozaki, Jinhai Li, Hiroki Akazawa, Kazuya Kishimoto, Koji Kinoshita, Itaru Matsumura

**Affiliations:** Department of Hematology and Rheumatology, Faculty of Medicine, Kindai University, Osaka 589-8511, Japan; jinhai@med.kindai.ac.jp (J.L.); hiroro2114@med.kidai.ac.jp (H.A.); kazuya-k@med.kidai.ac.jp (K.K.); kkino@med.kidai.ac.jp (K.K.); imatsumura@med.kindai.ac.jp (I.M.)

**Keywords:** microscopic polyangiitis, MPA, antineutrophil cytoplasmic antibody, antineutrophil cytoplasmic antibody-associated vasculitis, kidney injury molecule-1, Kim-1

## Abstract

Background: Microscopic polyangiitis (MPA) is a type of necrotizing vasculitis that primarily affects small vessels and belongs to the spectrum of anti-neutrophil cytoplasmic antibody (ANCA)-associated vasculitides (AAVs). While previous studies have identified potential prognostic biomarkers, further research is needed to validate a reliable marker for risk stratification in clinical practice. Kidney injury molecule-1 (Kim-1), a transmembrane protein expressed on proximal tubular epithelial cells, has been implicated in tubular damage. This study investigated the potential of Kim-1 as a biomarker in MPA. Methods: Kidney biopsy tissues, along with urine and blood samples, were retrospectively analyzed from 52 MPA patients and compared to urine samples from 7 healthy controls. Global disease activity was assessed using the Birmingham vasculitis activity score (BVAS) and vasculitis damage index, while renal disease activity was evaluated using renal BVAS (BVAS-R). Results: Urinary Kim-1 levels were significantly elevated in MPA patients compared to healthy controls. Urinary Kim-1 was positively correlated with the Mayo Clinic Chronicity Score (MCCS) but not with the ANCA Kidney Risk Score (AKRiS), whereas tubular Kim-1 was associated with AKRiS but not with MCCS, indicating their distinct pathological significance. Higher tubular Kim-1 expression was observed in patients with elevated BVAS-R. Urinary Kim-1 levels correlated with proteinuria and were associated with the Mayo Clinic Chronicity Score (MCCS) and ANCA Kidney Risk Score (AKRiS) but not with glomerular lesion severity. Unlike C-reactive protein (CRP), neither urinary nor tubular Kim-1 predicted MPA recurrence. Conclusions: Urinary Kim-1 reflects histopathologic findings and renal impairment but does not predict systemic disease activity or recurrence in MPA, demonstrating its potential clinical utility as a biomarker for assessing chronic renal damage.

## 1. Introduction

Antineutrophil cytoplasmic antibodies (ANCAs) are autoantibodies directed against proteins found in neutrophilic granules and lysosomes, which can be translocated to the cell surface during immune activation. ANCA-associated vasculitis (AAV) is characterized by inflammation and the destruction of small- and medium-sized blood vessels and by the presence of ANCAs. ANCA-associated vasculitis includes microscopic polyangiitis (MPA), granulomatosis with polyangiitis (GPA), and eosinophilic granulomatosis with polyangiitis (EGPA) [1,2]. The diagnosis and treatment of ANCA disease are complicated by marked differences in its symptoms, signs, activity, chronicity, and severity among patients. Prompt diagnosis and the initiation of the appropriate immunosuppressive therapy are essential for life expectancy and renal outcome [3].

MPA is a multisystem autoimmune disease characterized by necrotizing vasculitis involving small blood vessels, and patients are older than those with GPA and EGPA [4]. The most common organ damage is kidney disease, which is often accompanied by gross hematuria and rapidly progressive glomerulonephritis (RPGN); the 60-month survival rate for MPA is 45–76%, which is lower than for the other subtypes of AAV, GPA, and EGPA, and the main factor affecting patient survival is the severity of the initial kidney dysfunction [5]. In addition, infection has been reported as a major cause of death in MPA patients, particularly in older individuals, and immunosuppressive therapy has been identified as a key risk factor for infectious complications [6,7]. Therefore, identifying markers of renal impairment in MPA is important for predicting prognosis and determining an appropriate course of treatment.

ANCAs are known to play an important role in the pathogenesis of vasculitis. Neutrophils exposed to inflammatory cytokines are primed and ANCAs bind to cell-surface autoantigens, resulting in neutrophil activation. Activated neutrophils bind to vascular endothelium and induce tissue damage through the abnormal production of inflammatory cytokines and the extracellular release of neutrophil extracellular traps [8].

ANCAs have similarly been implicated in the pathogenesis of ANCA-associated glomerulonephritis. Its pathology is characterized by semilunar bodies with necrosis without deposition of immune complexes, which often destroy Bowman’s cysts and induce tubulointerstitial inflammation [9].

Unfortunately, however, the value of ANCA titers for predicting disease activity in vasculitis and nephritis is controversial. In particular, it is known that myeloperoxidase anti-neutrophil cytoplasmic antibody (MPO-ANCA) titer and disease activity diverge [10], and that the divergence can be explained by differences in MPO-ANCA epitopes and plateau affinity [11]. In addition, 2–3% of patients with pauci-immune necrotizing glomerulonephritis may be ANCA-negative but have pathological findings and clinical syndromes similar to ANCA-associated nephritis and ANCA-negative disease reported as pauci-immune necrotizing glomerulonephritis [12].

Kidney injury molecule-1 (Kim-1) is a transmembrane protein present on the surface of proximal tubular epithelial cells and is not expressed in normal kidneys [13]. We found that urinary and tubular Kim-1 expression is elevated in active lupus nephritis [14]. Urinary Kim-1 levels were also correlated with proteinuria and tubular damage [15]. Lieberthal et al. reported that Kim-1 was elevated in abnormal urine, and the monocyte chemoattractant protein-1 (MCP-1) protein/creatinine ratio was highest in AAV. This suggests that renal impairment and proteinuria are drivers of marker elevation, rather than unrecognized inflammation. There also appeared to be no difference in marker levels among patients with active non-renal disease stratified by hematuria or proteinuria, but the power to make such an assessment was weak [15]; thus, whether Kim-1 expression is a useful biomarker as a predictor of disease activity and the recurrence of AAV is unknown.

In the present study, we evaluated Kim-1 expression in the kidney tissue and urine of MPA patients. Given its established role in renal tubular injury and its association with proteinuria and tubular damage, we investigated whether Kim-1 could serve as a useful biomarker for renal impairment and prognosis in MPA patients.

## 2. Materials and Methods

### 2.1. Patients

This retrospective study was conducted at Kindai University Hospital between October 2011 and November 2023. The study was approved by the Institutional Review Board (approval No. 26-161), and all procedures were performed in accordance with national ethical guidelines and the Declaration of Helsinki.

Informed consent was obtained from all patients, including approval for the use of clinical data in research, academic presentations, and publications. Patients were informed that consent could be withdrawn at any time, with clear instructions provided. For deceased patients or those lost to follow-up, an opt-out procedure was implemented according to institutional ethical regulations, ensuring that patients or their families had the opportunity to decline participation. All data were anonymized before analysis to ensure patient confidentiality.

The patient selection process is summarized as follows: Of 132 patients suspected of MPA, all were initially evaluated based on the 2012 Revised Chapel Hill Consensus Conference (CHCC) criteria at the time of data collection. To ensure alignment with the latest international classification standards, we subsequently confirmed that all included patients also met the 2022 ACR/EULAR classification criteria for microscopic polyangiitis (MPA) [16], without altering the composition of the study cohort. Among these, 56 underwent kidney biopsy at diagnosis. Of these, two patients were without renal pathological findings, one patient began vasculitis treatment without renal involvement before biopsy, and four patients lost to follow-up due to transfer during the study period were excluded, resulting in a final cohort of 52 MPA patients. All enrolled patients were followed until the end of the 2-year observation period or until death (Supplementary Figure S1).

Healthy controls were recruited from individuals who consented to participate in the study, had normal urinalysis and an estimated glomerular filtration rate (eGFR) > 60 mL/min/1.73 m^2^. A total of 17 healthy controls (median age 70 [IQR: 60–76] years; 6 males [35.3%] and 11 females [64.7%]) were included. No significant differences in age and gender were observed between the healthy controls and the MPA group. Unlike the MPA patients, the healthy controls were not followed longitudinally, and urine samples were collected at a single time point to assess baseline urinary Kim-1 levels. Therefore, no subsequent clinical outcomes or renal function changes were monitored in this group. All procedures and the data anonymization were performed in accordance with national ethical guidelines.

### 2.2. Urine Collection for Determination of Biomarkers

Samples were reviewed and approved for use by the Kinki University Standing Committee for Clinical Research based on the Declaration of Helsinki (approval No. 26-161). Normal urine was obtained from healthy volunteers. For biochemical tests, 24 h urine and blood samples were taken before kidney biopsy, and clinical follow-up urine and serum samples were taken every 6 months for 2 years.

Protein, creatinine, and urinary erythrocytes of urine samples were stored at −80 °C, and serological parameters (blood urea nitrogen [BUN], serum creatinine [sCr], C-reactive protein [CRP], erythrocyte sedimentation rate [ESR], and MPO-ANCA) were measured in the Biochemistry Section of Kinki University Hospital. All urine samples for marker measurement were handled in the same manner. Urine samples were taken on the morning of the consultation day at 6–7 months after kidney biopsy. Urine samples were collected in sterile cups, aliquoted into 1.5 mL sterile non-pyrogenic cryo tubes, and frozen at −80 °C. Urinary protein concentrations were measured by pyrogallol red assay.

### 2.3. Renal Morphological Damage

Renal biopsy specimens were fixed in 10% buffered formalin for 12 h, dehydrated, and embedded in paraffin. They were sectioned by standard procedures, stained with periodate Schiff, and scored in a blinded fashion by two specialists with extensive expertise in autoimmune renal pathology. The minimum number of glomeruli in the renal biopsy specimens included in the study was 14. Sclerosis and crescents were calculated as a percentage of the total number of glomeruli. A blinded renal pathologist scored tubular damage as atrophy and fibrosis according to four categories: 0, none; 1, mild (including <25% of interstitium); 2, moderate (25–50% of interstitium); and 3, severe (>50% of interstitium). CD68+ cells and tubular KIM-1 in macrophages were assessed by immunoperoxidase staining of snap-frozen 4 μm sections. The counts were counted, and the average was scored. The primary monoclonal antibodies were CD68 hybridoma culture supernatant (HB 198; American Type Culture Collection, Manassas, VA, USA) and TIM-1 (R&D Systems, Minneapolis, MN, USA). Based on these results, scoring was performed using the Mayo Clinic Chronicity Score (MCCS) [17], which comprises glomerulosclerosis, interstitial fibrosis, tubular atrophy, and arteriosclerosis, as well as the ANCA Kidney Risk Score (AKRiS) [18], which includes the parameters normal glomeruli, tubular atrophy, and interstitial fibrosis, and estimated glomerular filtration rate. Additionally, the histopathological classification proposed by Berden et al. [19] for ANCA-associated glomerulonephritis was also evaluated, categorizing lesions into focal, crescentic, mixed, and sclerotic subtypes.

### 2.4. Measurement of Urinary Kim-1 Levels

Rat anti-mouse TIM-1 monoclonal antibody (R&D Systems) was used at 1 mg/mL to measure Kim-1 (ng/24 h) in urine from patients and healthy subjects. Samples were incubated overnight at 4 °C in carbonate/bicarbonate buffer (pH 9.6); after blocking with 2% bovine serum albumin (BSA), the samples and recombinant mouse TIM-1 (R&D Systems, Minneapolis, MN, USA) were incubated as standard for 2 h. The plates were then incubated with 0.1 mg/mL biotinylated goat anti-mouse TIM-1 (R&D Systems, Minneapolis, MN, USA) and streptavidin-HRP (Chemicon International, Billerica, MA, USA) for 1 h at room temperature. Each plate was developed using tetramethylbenzidine substrate and optical density was read at 450 nm; the detection limit of ELISA was 156.2 pg/mL.

### 2.5. Clinical Follow-Up

The initial assessment was performed on the day of renal biopsy, at which time we defined the baseline. After initial evaluation, all patients were followed for 2 years. Clinical management was determined by individual rheumatologists and was unaffected by the study. Patients received corticosteroids and/or cyclophosphamide, rituximab, or azathioprine as immunosuppressive treatments according to published protocols. No patient stopped treatment because of side effects. Relapse was defined as intensification or alteration due to an inadequate response to current therapy. However, treatment modifications were not necessarily driven by renal worsening alone; other clinical factors such as fever or an increase in MPO-ANCA levels also contributed to treatment decisions.

Global disease activity was evaluated using the Birmingham Vasculitis Activity Score (BVAS) and the Vasculitis Damage Index (VDI). For renal lesions, renal disease activity was assessed using renal BVAS (BVAS-R), which is a score comprising hypertension, proteinuria, hematuria, and serum creatinine. BVAS-R ranges from 0 to a maximum of 12.

### 2.6. Statistical Analysis

All statistical analyses were performed using the JMP Pro 18 software package (SAS Institute Inc., Cary, NC, USA). Continuous variables were summarized as medians and interquartile ranges (IQRs). Categorical variables were presented as percentages. The normality of continuous variables was assessed using the Shapiro–Wilk test. For correlation analyses, Spearman’s correlation coefficient was used to evaluate relationships between continuous variables, as it does not assume normality and is robust to non-normal distributions. Group comparisons for continuous variables across three or more categories were conducted using the Kruskal–Wallis test. A *p*-value of <0.05 was considered statistically significant. To further explore associations between variables, multivariable regression models were constructed. Ordinary least squares regression was applied to variables that followed a normal distribution, while generalized linear models were used for variables that did not meet the normality assumption. Comparisons of urinary KIM-1 levels between MPA patients and healthy controls were performed using the Mann–Whitney U test. To determine the optimal cut-off value for urinary KIM-1 in distinguishing MPA patients from healthy controls, receiver operating characteristic (ROC) curve analysis was conducted.

## 3. Results

### 3.1. Patient Characteristics

Table 1 shows patient characteristics, laboratory parameters, renal histopathology findings, and disease activity scores. The most frequent histological type was the focal type. However, the histological type was not correlated with the presence or absence of recurrence or with laboratory parameters.

### 3.2. Urinary Levels of Kim-1 in MPA Patients

**Figure 1:**
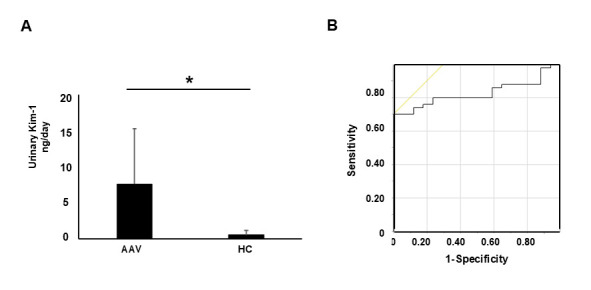
(**A**) Urinary Kim-1 levels in MPA patients and healthy controls. Asterisks (*) indicate a significant difference (*p* < 0.05). (**B**) Receiver operating characteristic (ROC) curve for urinary Kim-1.In panel (**B**), the black stepped line represents the receiver operating characteristic (ROC) curve, illustrating the diagnostic performance of urinary Kim-1. The yellow diagonal line represents the reference line for random classification.

Urinary Kim-1 levels (ng/day) were measured in the MPA patients and normal healthy volunteers. Urinary concentrations of Kim-1 (ng/g creatinine) standardized by urine creatinine were measured by ELISA in the same urine collected from all MPA patients at the baseline. Urinary Kim-1 levels in the MPA patients were significantly increased compared with levels in the healthy controls (5.0 (2.5–10.1) vs. 0.2 (0.1–0.5) ng/day; *p* < 0.05) (Figure 1A). The receiver operating characteristic (ROC) curve for urinary Kim-1 demonstrates its diagnostic performance in distinguishing MPA patients from healthy controls, with an optimal cut-off of 3.19 and an AUC of 0.82 (Figure 1B).

### 3.3. Correlations Between Urinary Kim-1 Level, Tubular Kim-1 Cell Count, Disease Activity, and Laboratory Parameters

Expression of tubular Kim-1 was confined to the apical side of the dilated tubules in the fibrotic areas of the kidneys of patients with active MPA, as we reported previously [14].

**Figure 2:**
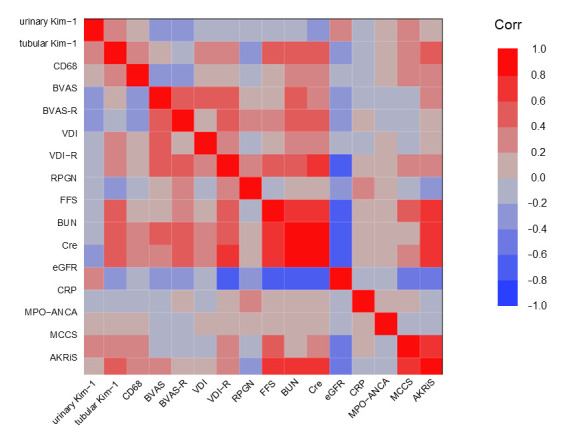
Heatmap representation of the correlation between tubular Kim-1, urinary Kim-1, and clinical/laboratory parameters in MPA patients. The color scale represents Pearson’s correlation coefficient (r), with red indicating positive correlations and blue indicating negative correlations.

Correlations between tubular Kim-1, urinary Kim-1, and clinical/laboratory parameters are summarized in Figure 2 (heatmap). The heatmap analysis revealed distinct correlation patterns between tubular Kim-1 and urinary Kim-1.

Urinary Kim-1 was positively correlated with MCCS (*p* = 0.02) but did not correlate with AKRiS. In contrast, tubular Kim-1 showed a significant correlation with AKRiS but not with MCCS, indicating a potential difference in the pathological significance of these markers.

Furthermore, tubular Kim-1, but not urinary Kim-1, was positively correlated with the five-factor score (FFS), blood urea nitrogen (BUN), serum creatinine (Cr), the estimated glomerular filtration rate (eGFR), and CD68-positive cell infiltration, suggesting its stronger association with renal injury severity and inflammatory cell infiltration. These findings highlight the differential clinical and pathological relevance of urinary and tubular Kim-1 in MPA-associated nephritis.

**Figure 3:**
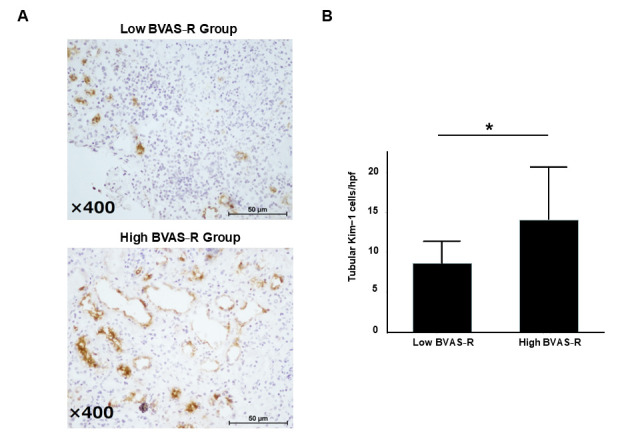
The relationship between tubular Kim-1-positive cells and BVAS-R. (**A**) Representative immunostained images of tubular Kim-1 expression in kidney biopsy specimens from MPA patients, categorized into the low BVAS-R group (BVAS-R < 10) and the high BVAS-R group (BVAS-R ≥ 10). (**B**) A quantitative comparison of tubular Kim-1-positive cell counts between the low and high BVAS-R groups, demonstrating a significantly higher expression in the high BVAS-R group (*p* < 0.05). Asterisks (*) indicate statistical significance.

We classified MPA patients with BVAS-R ≥ 10 as the high BVAS-R group and those with BVAS-R < 10 as the low BVAS-R group for the estimation of tubular Kim-1 expression. The high BVAS-R group had significantly increased tubular Kim-1 expression compared with the low BVAS-R group (12.7 (7.2–18.9) vs. 7.2 (6–10) cells/hpf; *p* < 0.05) (Figure 3). However, there was no difference in urinary Kim-1 levels between active and inactive patients, and BVAS-R could not predict urinary Kim-1 levels.

### 3.4. Changes in Kim-1 During Clinical Follow-Up

**Figure 4:**
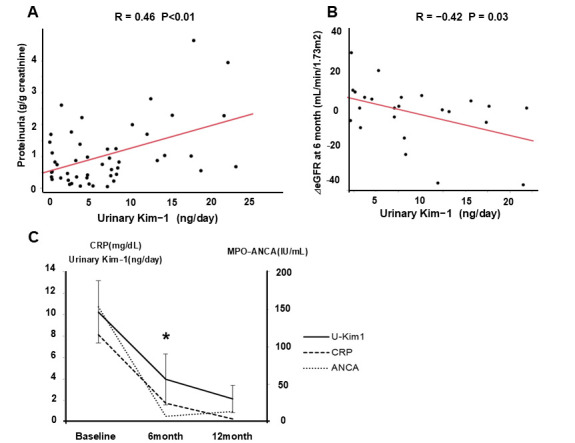
(**A**) Urinary Kim-1 at baseline was positively correlated with proteinuria (R = 0.46; *p* < 0.01), suggesting its potential role as a marker of tubular injury in MPA. The red line represents the regression line. (**B**) Urinary Kim-1 at baseline was also correlated with changes in eGFR at 6 months (R = 0.42; *p* = 0.03), indicating its potential predictive value for renal function decline. The red line represents the regression line. (**C**) Changes in urinary Kim-1 levels at 6 and 12 months after renal biopsy are shown. Kim-1 levels gradually decreased with treatment, similar to inflammatory markers such as CRP and ANCA, suggesting that urinary Kim-1 levels may reflect disease activity over time. Asterisks (*) indicate a statistically significant decrease in urinary Kim-1 from baseline to 6 months (*p* < 0.05).

Although urine protein and the estimated glomerular filtration rate (eGFR) at the baseline were not correlated, urinary Kim-1 at the baseline was correlated with proteinuria (R = 0.46; *p* < 0.01) (Figure 4A), and the change in eGFR at 6 months (R = 0.42; *p* = 0.03) (Figure 4B). However, urinary Kim-1 at the baseline did not predict the subsequent relapse of MPA. Figure 4C showed the changes in urinary Kim-1 at 6 and 12 months after renal biopsy in patients other than those who died or discontinued outpatient treatment. As shown in the figure, urinary Kim-1 decreased with the course of treatment, similar to laboratory parameters such as CRP and ANCA.

### 3.5. Relationship Between Urinary Kim-1 and Histological Parameters in MPA Patients

The urinary Kim-1 level was significantly associated with interstitial fibrosis (R = 0.34; *p* = 0.04), mesangial cell proliferation (R = 0.46; *p* < 0.01), and sclerotic lesions (R = 0.43; *p* < 0.01). The urinary Kim-1 level was not correlated with the severity of glomerular lesions including crescent formation.

**Figure 5:**
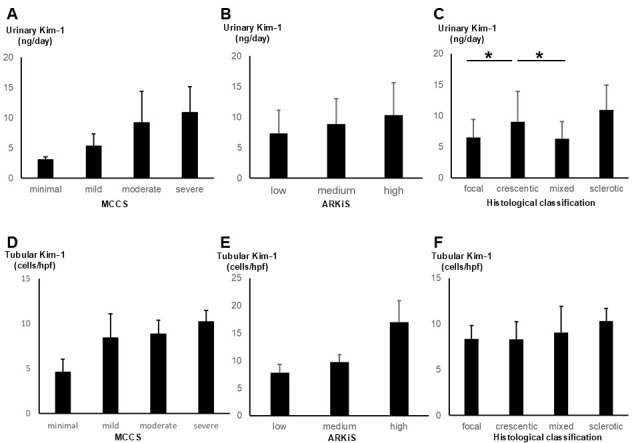
Urinary and tubular Kim-1 levels stratified by histopathological classification systems. (**A**–**C**) Urinary Kim-1 levels categorized according to (**A**) Mayo Clinic Chronicity Score (MCCS), (**B**) ANCA Kidney Risk Score (AKRiS), and (**C**) Berden’s histopathological classification. (**D**–**F**) Tubular Kim-1 levels categorized according to (**D**) MCCS, (**E**) AKRiS, and (**F**) Berden’s classification. Data are presented as mean ± standard deviation. Statistical comparisons were performed using Kruskal–Wallis test. Asterisks (*) indicate statistically significant differences in (**C**) (*p* < 0.05).

Urinary Kim-1 was correlated with MCCS (R = 0.30, *p* = 0.02); however, no significant differences were observed across severity classifications (Figure 5A). Similarly, urinary Kim-1 levels categorized according to AKRiS did not show significant differences across classifications (Figure 5B). Figure 5C shows the cell expression of urinary Kim-1 levels in relation to the histological classification of Berden et al. [19]. The crescent type was associated with significantly greater expression of urinary Kim-1 than the focal and mixed types (*p* = 0.04 and *p* = 0.04, respectively).

Tubular Kim-1 was correlated with MCCS but did not show significant differences across classifications (Figure 5D). Similarly, tubular Kim-1 levels categorized by AKRiS did not show significant differences (Figure 5E). No significant differences were observed in tubular Kim-1 levels among the histological classifications (Figure 5F). 

Table 2 shows the correlation between tubular Kim-1 cell count and histological findings. Tubular Kim-1-positive cell count correlated significantly with the percentage of total crescents formed (R = 0.40; *p* < 0.05) and the percentage of cellular crescents formed (R = 0.42; *p* < 0.05). The tubular Kim-1 cell count did not correlate with the percentage of fibrous crescents. There was also no correlation between the tubular Kim-1-positive cell count and mesangial cell proliferation or sclerotic lesion scores, the grade of stromal invasion, or the percentage area of CD68 expression.

To further investigate the relationship between urinary Kim-1 and renal histopathology, multivariable regression analyses were performed. Given the limited sample size (n = 52), only two additional variables were included alongside urinary Kim-1 to maintain statistical stability. CRP and MPO-ANCA were selected as covariates based on previous reports indicating that they reflect disease activity in vasculitis and influence renal prognosis [20]. In the multivariable analysis, urinary Kim-1 remained an independent predictor of MCCS (β = 0.102, 95% CI: 0.017–0.187, *p* = 0.019). In contrast, neither CRP nor MPO-ANCA were significantly associated with MCCS in the multivariable model (Table 3). These results suggest that urinary Kim-1 may serve as a biomarker reflecting renal chronicity.

Furthermore, we examined whether urinary Kim-1 was associated with renal function decline. In the univariate analysis, urinary Kim-1 was significantly correlated with ΔeGFR at 6 months (R = −0.42, *p* = 0.03). In the multivariable generalized linear model, urinary Kim-1 remained an independent predictor of ΔeGFR decline (β = −1.100, 95% CI: −2.142–0.059, *p* = 0.039). Similarly, CRP and MPO-ANCA were incorporated as explanatory variables based on their known associations with vasculitis severity and renal prognosis. However, neither CRP nor MPO-ANCA were significantly associated with ΔeGFR in the model. These findings indicate that urinary Kim-1 may not only reflect renal pathology but may also predict subsequent renal function decline.

### 3.6. Clinical Course and Predictors of Relapse

During the 2-year observation period, 13 patients (25%) died, and the 2-year survival rate was 75.0%. The most common cause was infection, followed by cardiovascular events and malignancy. Because the two patients who died of cardiovascular events died at another hospital or at home, it is not clear whether these deaths were due to MPA. None of the patients died of alveolar hemorrhage. In total, 38 patients (66%) had no recurrence for 2 years, and none of them developed end-stage renal disease requiring dialysis. When relapse occurred, steroids were increased in all cases, and immunosuppressants were changed in 4 of 14 patients (28.6%).

Urinary Kim-1 or tubular Kim-1 at baseline did not predict the subsequent recurrence of MPA, unlike CRP (Table 4). MPO-ANCA at baseline also failed to predict MPA relapse. However, CRP could predict relapse in MPA with an odds ratio of 38.8. The cut-off value was 9.9 mg/dL.

## 4. Discussion

Several papers have reported that tubular Kim-1 expression correlates with the degree of nephropathy in all inflammatory renal diseases [21]. Urinary Kim-1 is also increased in all inflammatory diseases in association with the degree of nephropathy [22,23,24], but not in hypertensive nephropathy or diabetic nephropathy [25]. The aim of the current study was to determine the utility of urinary Kim-1 levels to evaluate glomerular and tubular injury in histopathological examination as the renal disease activity in AAV patients. Our findings demonstrate that Kim-1 expression may be a useful biomarker for assessing renal damage but does not predict relapse.

First, we confirmed previous findings that urinary Kim-1 levels are higher in active MPA patients with AAV compared to healthy individuals. Furthermore, we found that urinary Kim-1 correlated with MCCS, whereas tubular Kim-1 was associated with AKRiS in MPA patients. Previously, we demonstrated that urinary and tubular Kim-1 levels are elevated in systemic lupus erythematosus (SLE) patients with lupus nephritis (LN) and are closely associated with renal disease severity [14]. In the present study, we observed similar findings in patients with AAV. While MCCS is recognized as a marker of chronic changes, AKRiS is known to predict ESRD [26]. These findings suggest that urinary Kim-1 may provide a more comprehensive assessment of chronic renal injury compared to tubular Kim-1.

Given these results, our study complements previous findings by incorporating AKRiS and MCCS, two recent histopathological classification systems, to refine the analysis of urinary Kim-1 in MPA-specific renal pathology. While previous studies have broadly examined urinary Kim-1 in CKD populations, our study focuses on MPA and its pathological characteristics, contributing additional perspectives on disease-specific renal involvement.

Moreover, the number of tubular Kim-1-positive cells also correlated with the degree of cellular crescent formation in LN patients. Ding et al. reported that urinary Kim-1 levels were correlated with the pathological features of tubular atrophy in LN and were significantly elevated in the presence of interstitial inflammatory lesions [27]. The reduction in urinary Kim-1 levels, alongside CRP and ANCA, during the follow-up period with treatment indicates that urinary Kim-1 is not solely a marker of irreversible chronic lesions. Furthermore, urinary Kim-1 levels were found to correlate with ΔGFR at 6 months. Thus, baseline urinary Kim-1 levels may reflect interstitial nephritis, as indicated by histological findings, as well as renal function during follow-up.

Second, we evaluated whether urinary and tubular Kim-1 expression, CRP, and MPO-ANCA titer at baseline are useful tools as predictors of relapse from baseline during follow-up. However, urinary and tubular Kim-1 levels did not predict MPA relapse, whereas CRP was a significant predictor. Unfortunately, we could not find a correlation between urinary Kim-1 levels and MPO-ANCA titers in patients who had relapsed, even though CRP was predictive for relapse during follow-up. These results suggest that Kim-1 expression primarily reflects renal involvement in MPA, whereas CRP is indicative of systemic vasculitis activity.

Renal biopsy is the gold standard for evaluating the degree of renal involvement in MPA; however, it is invasive and therefore not performed routinely. In the current study, we demonstrated that urinary Kim-1 has potential as a useful biomarker for the estimation of renal damage such as cellular crescent formation and interstitial inflammation. However, this study was limited in that it was retrospective, performed at a single center, and included only a small number of patients, which may have affected the statistical power of the multivariable analysis.

## 5. Conclusions

In conclusion, Kim-1 expression is elevated in MPA patients with renal impairment and is closely associated with the severity of renal disease. The present results demonstrate that urinary Kim-1 correlates with markers of chronic renal damage, particularly MCCS, while tubular Kim-1 is associated with AKRiS, indicating their distinct pathological significance. Furthermore, urinary Kim-1 levels were found to correlate with renal function decline over time, suggesting their potential role in monitoring disease progression. However, urinary and tubular Kim-1 did not predict the relapse of MPA, whereas CRP was identified as a significant predictor of recurrence. The most important conclusion from the current study is that urinary Kim-1 serves as a biomarker that reflects histopathological findings and renal impairment but does not predict systemic disease activity or recurrence in MPA patients.

## Figures and Tables

**Table 1:** Characteristics, laboratory parameters, renal histopathology findings, and disease activity.

Characteristics		Laboratory Parameters	
Female, %	78.8	BUN, mg/dL	20 (15–29.8)
Age, year	73 (65–78)	Serum creatinine, mg/dL	1.0 (0.7–1.7)
Duration, day	60 (45–106)	eGFR, mL/min/1.73 m^2^	43 (24.5–71.3)
		CRP, mg/dL	8.5 (3.7–13.7)
AGN classification		ESR, mm/h	72 (61.8–101.5)
Focal, n	27	MPO-ANCA, U/mL	122.3 (52.3–224.8)
Crescentic, n	5	Proteinuria, g/day	1.0 (0.5–1.7)
Mixed, n	15	Urinary RBCs/hpf	61.1 (28.7–243.2)
Sclerotic, n	4	urinary Kim-1, ng/days	0.5 (0.2–0.9)
MCCS		tubular Kim-1, cells/hpf	7.8 (6.5–11)
minimal, n	5	CD68, c/hpf	8.2 (6.5–11)
mild, n	21		
moderate, n	17	**Disease activity**	
severe, n	9	BVAS	12 (8–14)
AKRiS		BVAS-R	7 (5–10)
low, n	38	VDI	2 (1–3)
medium, n	11	VDI-R	1 (1–2)
high, n	3	clinical grade category of RPGN	1 (1–2)
very high, n	0	FFS	2 (1–2)

**Table 2:** Disease activity and the expression of tubular Kim-1 in MPA.

	Correlation Coefficient	*p*-Value
Mesangial proliferation (0–2)	0.09	0.80
Total glomerular sclerosis (%)	0.32	0.09
Segmental sclerosis (%)	0.14	0.44
Global sclerosis (%)	0.24	0.18
Total crescents, %	0.42	<0.05
Cellular crescents, %	0.42	<0.05
Fibrous crescents, %	0.14	0.42
Interstitial infiltration (0–3)	0.07	0.69
CD68 (cells/hpf)	0.14	0.46

**Table 3:** Multivariable Regression Analyses of Urinary Kim-1, Disease Activity Markers, and Kidney Outcomes.

Outcome	Variable	Regression Coefficient (β) (95% CI)	*p* Value
MCCS	urinary Kim-1	0.102 (0.017, 0.187)	0.019
	CRP	0.026 (−0.071, 0.122)	0.596
	MPO-ANCA	−0.002 (−0.006, 0.003)	0.477
ΔeGFR	urinary Kim-1	−1.100 (−2.142, −0.0591)	0.039
	CRP	−0.388 (−1.206, 0.431)	0.342
	MPO-ANCA	−0.000 (−0.045, 0.037)	0.841

**Table 4:** Relationship between relapse and laboratory parameters or Kim-1.

	OR	*p*-Value	Cut-Off
CD68, cell/HPF	11.6	0.16	-
Tubular Kim-1, cell/HPF	3.5	0.57	-
Urinary Kim-1, ng/g creatinine	2.08	0.57	-
CRP, mg/dL	38.8	<0.05	9.9
MPO-ANCA, U/mL	15.8	0.13	-

## Data Availability

The data presented in this study are available on request from the corresponding author due to privacy.

## Supplementary Materials

The supporting information can be downloaded at: https://www.mdpi.com/article/10.3390/cimb47030196/s1.

## Abbreviations

The following abbreviations are used in this manuscript:

MPA	microscopic polyangiitis
Kim-1	kidney injury molecule-1
BUN	blood urea nitrogen
eGFR	estimated glomerular filtration rate
CRP	C-reactive protein
ESR	erythrocyte sedimentation rate
MPO-ANCA	myeroperoxidase antineutrophil cytoplasmic antibody
RBC	red blood cell
AGN	acute glomerulonephritis
MCCS	Mayo Clinic Chronicity Score
AKRiS	ANCA Kidney Risk Score
BVAS	Birmingham Vasculitis Activity Score
BVAS-R	renal Birmingham Vasculitis Activity Score
VDI	Vasculitis Damage Index
VDI-R	renal Vasculitis Damage Index
RPGN	rapidly progressive glomerulonephritis
FFS	five-factor score
