# Supplementary material for: Urinary Kim-1 Correlates with Interstitial Nephritis Activity in Patients with Microscopic Polyangiitis
Source: Curr Issues Mol Biol. 2025 Mar 16;47(3):196. doi: 10.3390/cimb47030196 (PMC11941514; doi:10.3390/cimb47030196)
Supplement: Supplementary file 1 [file cimb-47-00196-s001.zip › cimb-3428981-supplementary.pdf]

Supplementary Figure

Supplementary Figure 1

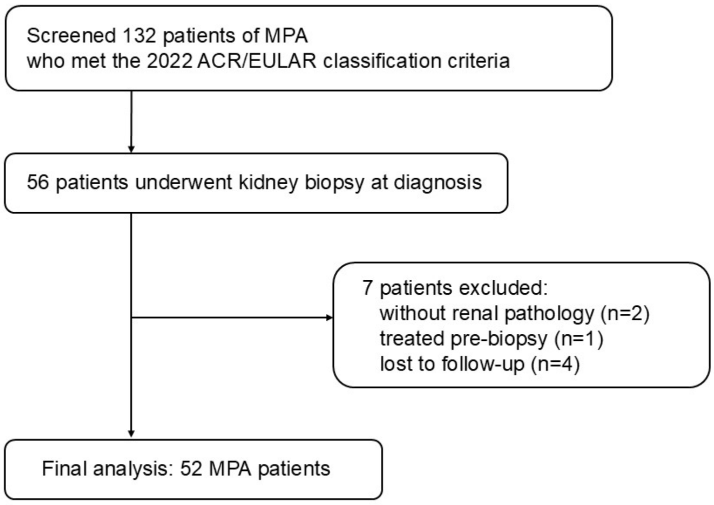

Supplementary Figure 1 . Flowchart of patient selection.
